# ESR Essentials: imaging in fibrotic lung diseases—practice recommendations by the European Society of Thoracic Imaging

**DOI:** 10.1007/s00330-024-11054-2

**Published:** 2024-09-07

**Authors:** Anna Rita Larici, Juergen Biederer, Giuseppe Cicchetti, Tomas Franquet Casas, Nick Screaton, Martine Remy-Jardin, Anagha Parkar, Helmut Prosch, Cornelia Schaefer-Prokop, Thomas Frauenfelder, Benoit Ghaye, Nicola Sverzellati

**Affiliations:** 1 Department of Radiological and Hematological Sciences, Catholic University of the Sacred Heart, Rome, Italy; 2 Department of Diagnostic Imaging and Oncological Radiotherapy, Advanced Radiology Center, ‘A. Gemelli’ University Polyclinic Foundation IRCCS, Rome, Italy; 3 Diagnostic and Interventional Radiology, University Hospital, Heidelberg, Germany; 4 Translational Lung Research Center Heidelberg (TLRC), Member of the German Center for Lung Research (DZL), Heidelberg, Germany; 5 University of Latvia, Faculty of Medicine, Riga, Latvia; 6 Christian-Albrechts-Universität zu Kiel, Faculty of Medicine, Kiel, Germany; 7 Department of Radiology, Hospital de Sant Pau, Barcelona, Spain; 8 Department of Radiology, Royal Papworth Hospital NHSFT, Cambridge, United Kingdom; 9 IMALLIANCE-Haut-de-France, Valenciennes, France; 10 Department of Thoracic Imaging, University of Lille, Lille, France; 11 Radiology Department, Haraldsplass Deaconess Hospital, Bergen, Norway; 12 Department of Clinical Medicine, Faculty of Medicine and Dentistry, University of Bergen, Bergen, Norway; 13 Department of Radiology, Medical University of Vienna, Vienna, Austria; 14 Radiology, Meander Medical Centre Amersfoort, Amersfoort, Netherlands; 15 Department of Radiology, Nuclear Medicine and Anatomy, RadboudUMC, Nijmegen, Netherlands; 16 Diagnostic and Interventional Radiology, University Hospital Zurich, University Zurich, Zurich, Switzerland; 17 Department of Radiology, Cliniques Universitaires St-Luc, Catholic University of Louvain, Brussels, Belgium; 18 Scienze Radiologiche, Department of Medicine and Surgery, University of Parma, Parma, Italy

**Keywords:** Pulmonary fibrosis, Tomography (x-ray computed), Lung diseases (interstitial), Diagnostic imaging, Disease progression

## Abstract

**Abstract:**

Fibrotic lung diseases (FLDs) represent a subgroup of interstitial lung diseases (ILDs), which can progress over time and carry a poor prognosis. Imaging has increased diagnostic discrimination in the evaluation of FLDs. International guidelines have stated the role of radiologists in the diagnosis and management of FLDs, in the context of the interdisciplinary discussion. Chest computed tomography (CT) with high-resolution technique is recommended to correctly recognise signs, patterns, and distribution of individual FLDs. Radiologists may be the first to recognise the presence of previously unknown interstitial lung abnormalities (ILAs) in various settings. A systematic approach to CT images may lead to a non-invasive diagnosis of FLDs. Careful comparison of serial CT exams is crucial in determining either disease progression or supervening complications. This ‘Essentials’ aims to provide radiologists a concise and practical approach to FLDs, focusing on CT technical requirements, pattern recognition, and assessment of disease progression and complications. Hot topics such as ILAs and progressive pulmonary fibrosis (PPF) are also discussed.

**Key Points:**

- *Chest CT with high-resolution technique is the recommended imaging modality to diagnose pulmonary fibrosis*.
- *CT pattern recognition is central for an accurate diagnosis of fibrotic lung diseases (FLDs) by interdisciplinary discussion*.
- *Radiologists are to evaluate disease behaviour by accurately comparing serial CT scans*.

## Key recommendations

- Chest CT with high-resolution technique is the recommended imaging modality to correctly recognise signs, patterns, and distribution of pulmonary fibrosis. A slice thickness of ≤ 1.5 mm and a high-resolution reconstruction algorithm are the basic requirements for a high-quality technique (Level of evidence: low).
- The accurate interpretation of CT pattern, along with clinical and laboratory data, often leads to a non-invasive diagnosis of specific fibrotic lung diseases (FLDs). Biopsy is recommended for cases with indeterminate radiologic-clinical features or in case of consequences for therapeutic decision-making (Level of evidence: low).
- FLDs may show progressive behaviour and reduced survival. An early diagnosis of fibrosis and prompt identification of disease progression are crucial for starting antifibrotic treatment for patients with idiopathic pulmonary fibrosis (IPF), as well as for non-IPF patients showing a progressive phenotype (Level of evidence: low). Careful comparison with previous CT examinations is essential to assess progression.

## Introduction

Interstitial lung diseases (ILDs) encompass a wide range of different entities, including idiopathic and secondary forms, with a variable degree of inflammation and fibrosis. Predominant fibrotic phenotype diseases, namely fibrotic lung diseases (FLDs), may have a progressive behaviour and worse prognosis [1], with idiopathic pulmonary fibrosis (IPF) being the prototype. Non-IPF diseases also may progress over time (e.g., progressive pulmonary fibrosis, PPF); identifying this group of patients is crucial as they may benefit from antifibrotic therapies like IPF [2]. FLDs are diagnosed in the appropriate clinical setting by interdisciplinary discussion based on radiological and/or histological patterns, as defined by current international guidelines [3]. Chest computed tomography (CT) with high-resolution technique plays an essential role in the identification of signs of lung fibrosis as well as in the assessment of disease progression and complications. Subtle interstitial lung abnormalities (ILAs) incidentally identified on CT also have the potential to worsen over time [4]. Application of quantitative CT methods has demonstrated promising results in evaluating disease progression, despite still not being routinely employed in clinical practice [5]. As part of the ‘ESR Essentials’ series, this paper provides concise and practical recommendations for general radiologists aimed to highlight essential imaging criteria for the diagnosis and management of FLDs.

## Practice recommendations

### High-risk patient categories

Radiologists may deal with patients affected by FLDs in different clinical scenarios. One scenario includes patients with respiratory symptoms (persistent dyspnoea, dry cough) that may present bibasilar Velcro-like crackles at physical examination and/or restrictive pattern at pulmonary function tests (PFTs), suspicious for ILD [6]. Another scenario encompasses patients at high risk of developing FLD due to different predisposing factors, including exposures, drugs, family history, and underlying diseases such as connective tissue diseases (CTDs). While IPF occurs more commonly in men and in people > 60 years of age, usually with a history of cigarette smoking, other FLDs (e.g., CTD-ILD, sarcoidosis) more frequently affect younger, female, and non-smoking patients [1]. FLDs may be potentially familial [7], and, when suspected, CT screening can be offered to first-degree relatives [8]. Radiologists should be aware of populations at high risk of developing FLDs.

### Imaging modalities

The imaging modality of choice for the detection and classification of ILDs is CT with high-resolution technique, which represents the most accurate non-invasive method for diagnosing pulmonary fibrosis. In this context, the role of chest X-ray is limited due to its low sensitivity and specificity, although in clinical practice it is used as a first-line imaging test in patients with respiratory symptoms [9]. Evidence of bilateral reticular or reticulonodular opacities on chest X-ray, associated with reduced lung volume, in the appropriate clinical setting, should lead radiologists to recommend a chest CT scan.

### CT acquisition: technical requirements

Consistent with the American Thoracic Society (ATS)/European Respiratory Society (ERS)/Japanese Respiratory Society (JRS)/Asociación Latinoamericana de Tórax (ALAT) guideline for the diagnosis of IPF, a noncontrast full lung coverage volumetric chest CT with high-resolution technique should be performed in supine position with arms above head, at deep inspiration [6]. Paired inspiratory/expiratory CT scans are not recommended as a routine protocol [10, 11]. The expiratory scan is recommended, especially upon initial assessment of ILDs, to recognise small airways’ involvement, which is commonly observed as air-trapping in hypersensitivity pneumonitis, rheumatoid arthritis and sarcoidosis [6, 10, 12]. In the follow-up, expiratory scans should be added on an individual basis, considering the patient’s symptoms, PFTs, and findings in the inspiratory CT scan. Being focused on functional information alone, the expiratory scans may be obtained at very low doses. An inspiratory scan in the prone position (sequential or volumetric) is optional, being useful if dependent lung atelectasis cannot be differentiated from interstitial changes.

Technically, multidetector CT is used with the shortest rotation time and high pitch, to reduce the acquisition time and motion artifacts. The standard tube voltage of 120 kVp may be adapted to the patient’s Body mass index (BMI) to keep the effective dose below 3 mSv [6]. Available tools to reduce radiation exposure, such as automatic exposure controls, organ dose modulation, postero-anterior adjustment of the field of view (FOV) and optimise image quality with advanced reconstruction algorithms (e.g., iterative or deep learning) are strongly encouraged. However, the use of low (< 1 mSv) or ultra-low dose (< 0.3 mSv) protocols is currently not recommended; they may be used only in selected cases and with advanced reconstruction algorithms. Images should be reconstructed at a slice thickness of ≤ 1.5 mm, with a high-resolution algorithm and a FOV adapted to full lung parenchyma coverage [6]. Reconstruction matrices beyond the standard 512 × 512 (pixel size 0.7 mm at a 35 cm FOV) with latest scanner technology (e.g., photon counting with voxel sizes down to 0.2 mm) are appreciated as far as achievable at acceptable noise level [13]. Table 1 summarises the CT technical requirements.

**Table 1:** Requirements for chest computed tomography (CT) with high-resolution technique

Acquisition parameters	
Scan mode	Volumetric multi-row detector acquisition
Tube voltage	120 kVp (100–140 adapted to BMI)
Rotation time	Shortest^a^ (≤ 0.5 s)
Table feed	Highest pitch^a^
Scan time	< 10 s
Effective dose	< 3 mSv
Intravenous contrast medium	None
Automatic exposure control (tube current/voltage modulation, automated kV selection)	Recommended
**Image reconstruction**	
Slice thickness	≤ 1.5 mm
Kernel	High-resolution
Reconstruction interval	Contiguous or overlapping (0.5–0.7 mm, depending on slice thickness)^b^
Advanced reconstruction algorithms	As far as applicable
Reconstruction matrix	Minimum 512 × 512
Minimum intensity projection (mIP)	Slab thickness 7–10 mm (for differentiating honeycombing versus bronchiectasis, visualisation of air trapping)

^a^ Shortest/highest at which the number of projections is not reduced

^b^ Contiguous reconstruction (no overlap) may be used at submillimetre slice thickness

### CT signs of pulmonary fibrosis and pattern recognition

In the appropriate clinical setting, the correct interpretation of the CT appearance may allow an accurate diagnosis of FLD, obviating the need for invasive tests [14].

#### Signs of pulmonary fibrosis

**Fig. 1:**
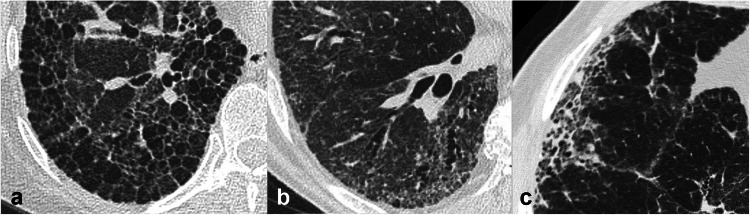
Computed tomography (CT) signs of pulmonary fibrosis. Honeycombing, characterised by destruction of lung parenchyma replaced by well-defined cystic spaces with thick walls, typically clustered in multiple layers in the subpleural region (**a**). Traction bronchiectasis, characterised by bronchial irregular dilatation in a background of intralobular fibrosis. Note the mild volume reduction of the right lower lobe, demonstrated by the posterior displacement of the major fissure (**b**). Fibrotic reticulation with irregular thickening of the interlobular and intralobular septa, associated with distal traction bronchiolectasis, in the subpleural region (**c**). Architectural distortion of the lung is present in all CT images

According to the Fleischner Glossary of Terms [15], the term fibrosis refers to a repair mechanism in which lung parenchyma is permanently replaced by connective tissue, causing remodelling, architectural distortion, and volume loss. Signs and patterns of pulmonary fibrosis useful to the interpretation of CT scans have been described (Fig. 1).

##### Honeycombing

Honeycombing represents the destruction of lung parenchyma replaced by well-defined cystic structures, typically clustered in the subpleural region. Honeycombing can be identified even in cases of a single layer of cysts, provided other signs of fibrosis are present [15]. Its presence in a basal and posterior location is the most specific sign associated with the usual interstitial pneumonia (UIP) pattern. The identification of honeycombing can be challenging if subpleural cysts are small and scanty and in the presence of traction bronchiectasis or paraseptal emphysema [16]. Correct identification establishes a prognosis since it reflects end-stage pulmonary fibrosis. Honeycombing can be present in IPF as well as in other conditions, such as fibrotic hypersensitivity pneumonitis (fHP), fibrotic sarcoidosis, and fibrotic nonspecific interstitial pneumonia (fNSIP).

##### Traction bronchiectasis and bronchiolectasis

Traction bronchiectasis consists of an irregular dilatation of the bronchial lumen associated with thickened, irregular bronchial walls, in the context of CT features of lung fibrosis; traction bronchiolectasis represents dilatation of bronchioles associated with fibrosis. Both represent the most persistent and important indices of the severity and prognosis of FLDs [17].

##### Architectural distortion

This term refers to the focal or diffuse disruption of the normal pulmonary anatomy (airway, vessels, and interstitium), usually associated with volume loss [15].

##### Reticular pattern

The term “reticular pattern” is due to fibrotic or non-fibrotic interstitial thickening at the level of the interlobular septa or the intralobular interstitium. Fibrotic reticulation consists of a fine or coarse reticular network that extends from the central peribronchovascular structures of the lobule to the interlobular septa. It may also be associated with traction bronchiectasis and bronchiolectasis, honeycombing, and architectural distortion.

#### Pattern recognition in pulmonary fibrosis

**Fig. 2:**
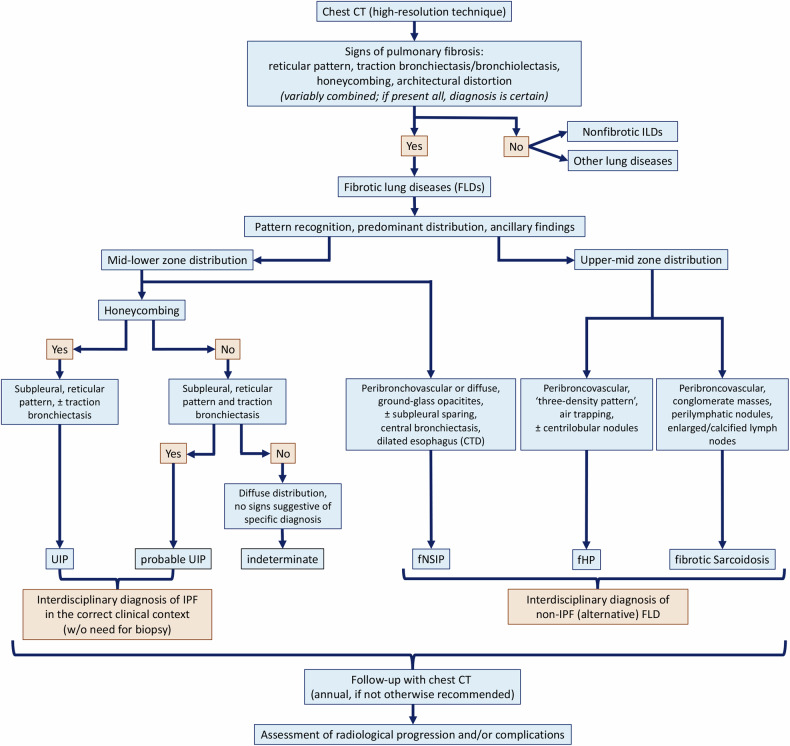
Diagnostic flowchart of fibrotic lung diseases (FLDs). The systematic approach to computed tomography (CT) analysis and interpretation leads to a non-invasive, accurate diagnosis of FLDs in the appropriate clinical setting by interdisciplinary discussion. CT, computed tomography; FLD(s), fibrotic lung disease(s); ILD(s), interstitial lung disease(s); CTD, connective tissue disease; UIP, usual interstitial pneumonia; fNSIP, fibrotic nonspecific interstitial pneumonia; fHP, fibrotic hypersensitivity pneumonitis; IPF, idiopathic pulmonary fibrosis

The pattern and distribution of fibrosis, as well as ancillary findings, are crucial in refining the diagnosis (Fig. 2).

##### Mid and lower zone distribution

**Fig. 3:**
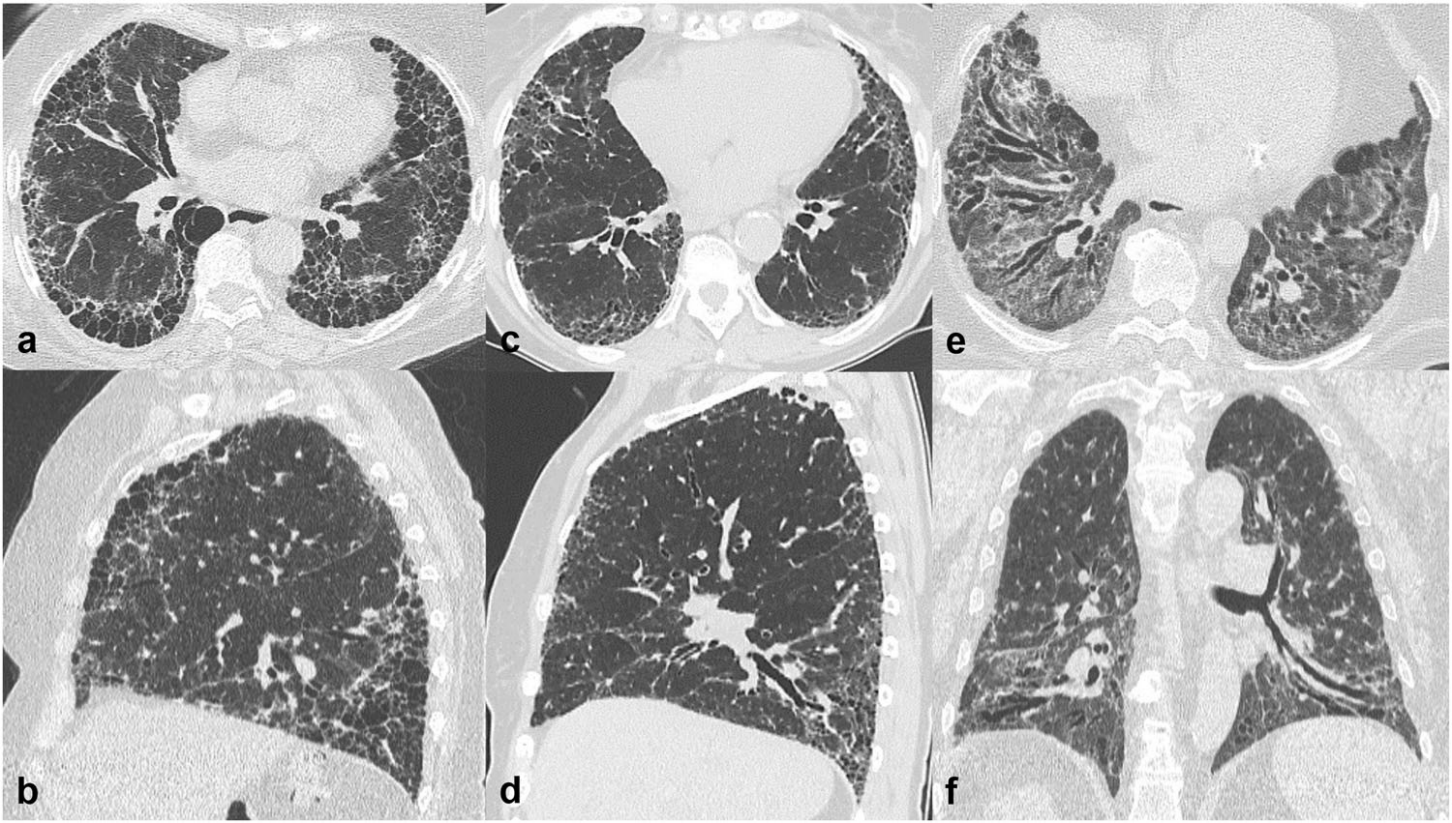
Computed tomography (CT) patterns of fibrotic lung diseases with mid-lower zone predominant distribution. Axial and sagittal CT images showing a usual interstitial pneumonia (UIP) pattern, characterised by honeycombing and reticular pattern with (or without) traction bronchiectasis, and a typical peripheral predominance in the lower lobes but also in the anterior region of the upper lobes (helical distribution) (**a**, **b**). Axial and sagittal CT images depicting a probable UIP pattern, characterised by a reticular pattern with distal traction bronchiectasis, with the same distribution as typical UIP but lacking honeycombing (**c**, **d**). Axial and coronal CT images of a fibrotic nonspecific interstitial pneumonia (fNSIP), with reticular pattern and traction bronchiectasis showing a typical peribronchovascular distribution. Note the volume reduction of the lower lobes (**e**, **f**)

In the UIP pattern fibrotic changes are characteristically peripheral, dorsal mid and lower zones predominant with traction bronchiectasis (in probable UIP) and/or honeycombing (in UIP). Definite and probable UIP patterns are associated with a high probability of a diagnosis of IPF in the correct clinical context [3] (Fig. 3). UIP may also be seen in other conditions (e.g., connective tissue disease, asbestosis, hypersensitivity pneumonitis) with or without ancillary CT features. fNSIP may be associated with a variable extent of ground-glass opacities (reflecting interstitial inflammation) and a more diffuse or peribronchovascular distribution than in UIP (Fig. 3).

##### Mid and upper zone distribution

**Fig. 4:**
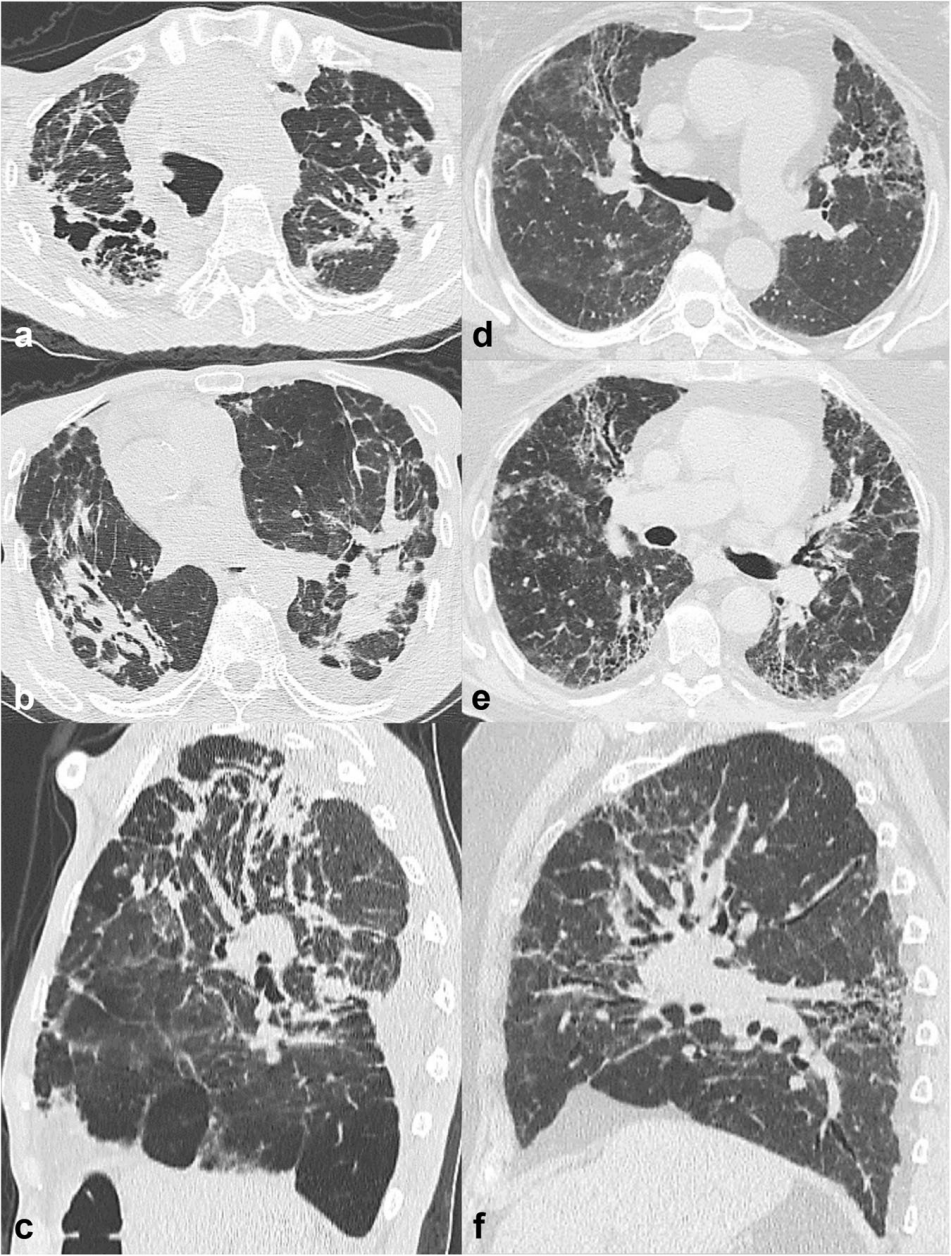
Computed tomography (CT) patterns of fibrotic lung diseases with upper-mid predominant distribution. Axial and sagittal CT images (**a**–**c**) showing peribronchovascular conglomerate masses in the upper lobes associated with parenchymal and airways distortion in the right upper lobe in a fibrotic sarcoidosis. Note the right upper lobe volume reduction and areas of mosaic attenuation in the mid-lower zones due to air trapping. Axial and sagittal CT images (**d**–**f**) demonstrating reticulation and mild traction bronchiectasis with a peribronchovascular predominant distribution, in a fibrotic hypersensitivity pneumonitis. Note the association with centrilobular ground-glass nodules, partially confluent, and a mild ‘three-density pattern’ in the upper-mid zones

In fibrotic sarcoidosis and fHP, fibrosis is most commonly peribronchovascular and located in the mid and upper zones. In both conditions, other CT findings supporting the underlying aetiology are present; the ‘three-density pattern’ (coexistence of ground-glass opacities, decreased lung attenuation and normal lung) is highly specific for fHP and facilitates differential diagnosis with IPF [12], while perilymphatic nodules, conglomerate peribronchovascular masses and enlarged or calcified nodes support the diagnosis of sarcoidosis (Fig. 4).

### Reading modalities

CT image reading should be performed by qualified radiologists with an appropriate level of training and expertise.

Since FLD distribution (craniocaudal gradient, involvement of the dorsobasal region, peripheral or peribronchovascular) is of large diagnostic importance, reconstruction and analysis of sagittal and coronal multiplanar reconstructions is strongly recommended. Maximum intensity projections (MIP) are advantageous for classifying the distribution of nodular opacities (random, perilymphatic and centrolobular). In the field of FLDs, minimum intensity projections are particularly helpful in differentiating traction bronchiectasis from honeycombing and in diagnosing the extent and distribution of lobular air trapping (Supplementary Fig. S1). Consistency in CT technique and image quality between serial CT scans is of paramount importance, and any variation should be accounted for.

### Progressive pulmonary fibrosis

**Fig. 5:**
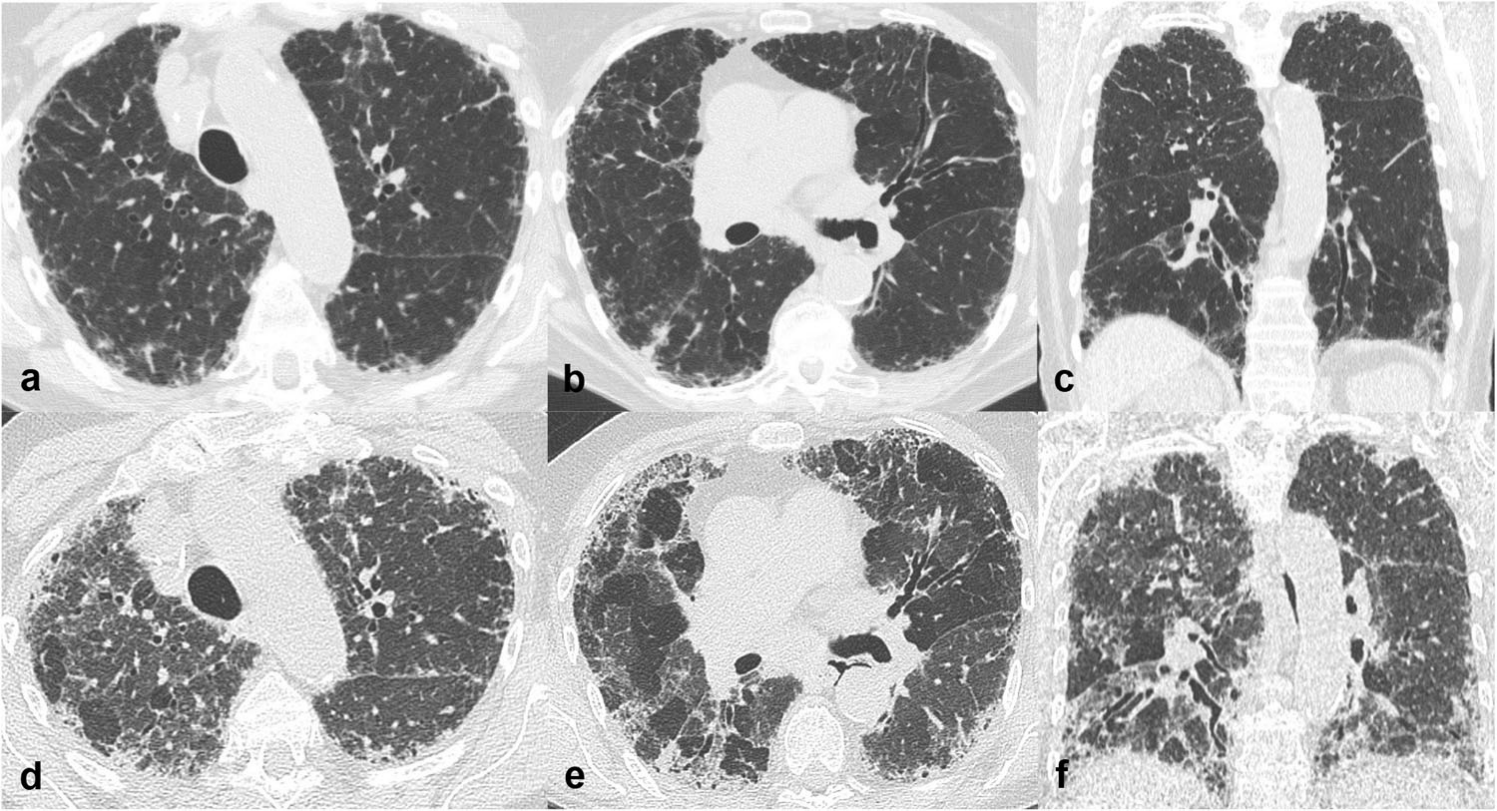
Progressive pulmonary fibrosis (PPF). Axial and coronal computed tomography (CT) images (**a**–**c**) showing mild signs of fibrosis without honeycombing in the subpleural areas bilaterally, with mild predominance in the lung bases, suggestive of a probable usual interstitial pneumonia (UIP) pattern in a patient with chronic exposure to pigeons. The multidisciplinary discussion provided a provisional diagnosis of fibrotic hypersensitivity pneumonitis (fHP). Due to worsening dyspnoea, the patient underwent a follow-up CT after 13 months. Axial and coronal CT images (**d**–**f**) demonstrate unequivocal disease progression with respect to the previous exam, with increased extent and coarseness of the irregular interstitial thickening and traction bronchiectasis, predominant at the periphery in the upper lobes and more extensive in the mid-lower lungs, associated with marked ‘three-density pattern’ (in inspiratory scans) and lung volume loss. A diagnosis of fHP with progressive behaviour was confirmed, and antifibrotic treatment was initiated

Progressive pulmonary fibrosis (PPF) is an evolving challenge within the FLD field, marked by worsening respiratory symptoms, functional decline, and radiological progression despite traditional pharmaceutical management. IPF is excluded from this group, being progressive by definition [3]. A progressive phenotype is observed in about 25% of FLDs other than IPF, underscoring the prevalence and impact of PPF [18]. Radiologists play a crucial role in diagnosing and monitoring PPF. The presence and severity of fibrotic changes on CT significantly correlate with disease progression and mortality [19]. Signs indicative of radiological progression include new or increased fibrotic features (e.g., new or increased coarseness of reticular abnormality, increased extent or severity of traction bronchiectasis or honeycombing, etc.) and increased volume loss [3] (Fig. 5). The advent of antifibrotic therapies offers hope, marking significant advancements in treating this challenging condition [2]. Ultimately, radiological assessment through CT is pivotal in the early diagnosis and management of PPF, making radiologists integral to the interdisciplinary approach required for optimal patient outcomes.

### Assessment of disease progression and follow-up

There is currently no guidance on the optimal use of follow-up CT, though annual follow-up is usually appropriate to rule out disease progression and complications. In clinical practice, disease progression is usually identified by the integration of clinical, functional, and radiological data.

**Fig. 6:**
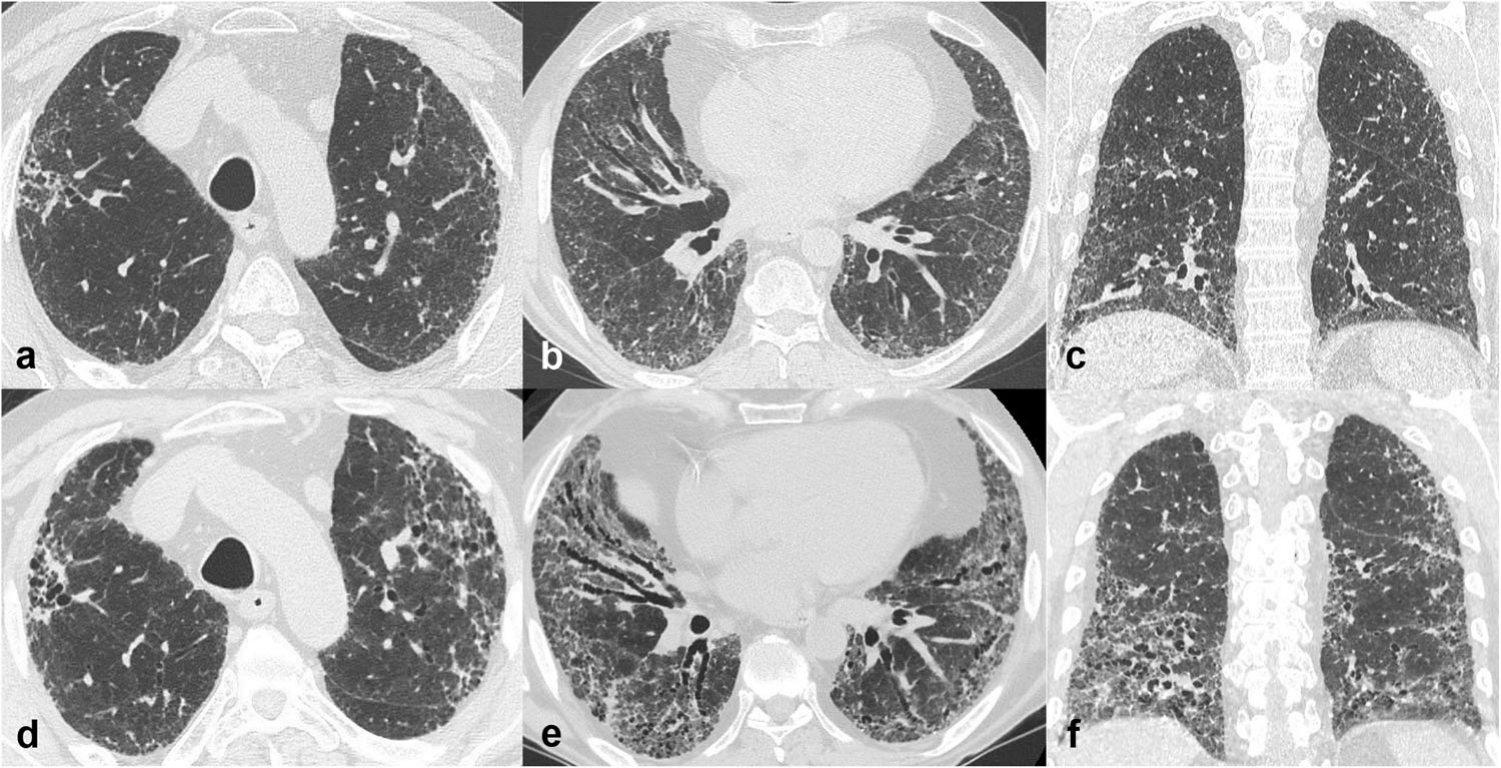
Progressive pulmonary fibrosis (PPF). Axial and coronal computed tomography (CT) images (**a**–**c**) showing mild reticulation with slight central traction bronchiectasis/bronchiolectasis, predominant in the lung bases, suggestive of a nonspecific interstitial pneumonia (NSIP) pattern in a patient with systemic sclerosis. Axial and coronal CT images (**d**–**f**) performed after 15 months due to worsening dyspnoea, demonstrate evident progression to fibrotic NSIP (fNSIP), with increased reticulation coarseness, central traction bronchiectasis and parenchymal distortion, predominant in the lung bases, and increased volume loss (**f**). Note that image analysis is conducted at the same anatomic levels to accurately assess disease progression. Antifibrotic treatment was started

Serial CT scans should be compared ‘side by side’ on any plane—axial, sagittal, coronal—to capture changes in the pattern or increased extent of fibrosis. In fact, individual FLDs may progress differently. UIP usually shows an increase in the extent of disease, whereas NSIP tends to remain stable in extent but displays changes in individual pattern (e.g., increased coarseness of reticular abnormality, increased traction bronchiectasis) (Fig. 6). Furthermore, radiologists must differentiate between disease progression and complications that might occur in subjects with lung fibrosis. There are no specific thresholds to define disease progression so that any change could be meaningful. Moreover, coexisting pulmonary emphysema should be promptly assessed as it might perturb the clinico-functional assessment.

Visual assessment of the longitudinal behaviour of lung fibrosis can be quite cumbersome and is prone to interobserver variability, especially if it is subtle [9]. There is commercially available software that allows for automatic quantification of various CT patterns, including the vasculature. While this might play a role in research and controlled studies, there is not yet a broad application in clinical practice. Equally recent research focused on the development and evaluation of deep learning-based software for pattern recognition and classification [20]. While this might play a larger role in the future, it is currently limited to research applications.

### Interstitial lung abnormalities

ILAs are defined as incidental, non-dependent subtle interstitial abnormalities detected in at least 5% of a lung zone (distinguished in upper, middle, and lower lung zones, as demarcated by the inferior wall of the aortic arch and the right inferior pulmonary vein), in individuals where ILD is not initially suspected [4]. ILAs are identified in up to 9% of smokers and in approximately 7% of non-smokers. Notably, their prevalence can reach up to 25% in lung cancer screening cohorts [21]. ILAs are categorised into non-subpleural ILAs, subpleural non-fibrotic ILAs, and subpleural fibrotic ILAs [4] (Supplementary Fig. S2). Non-subpleural ILAs generally show no progression, whereas fibrotic ILAs are known to progress and are associated with increased mortality [4]. The recent Fleischner Society white paper advises that individuals with ILAs and evidence of clinically significant disease should undergo further evaluation [4]. This is to ensure accurate diagnosis and appropriate treatment of any clinically significant ILD. Individuals with clinically insignificant ILAs should be monitored both clinically and radiologically for signs that might suggest an increased risk of progression, such as UIP or probable UIP patterns. For those with ILAs but without risk factors, expectant management is recommended [4].

### Complications

**Fig. 7:**
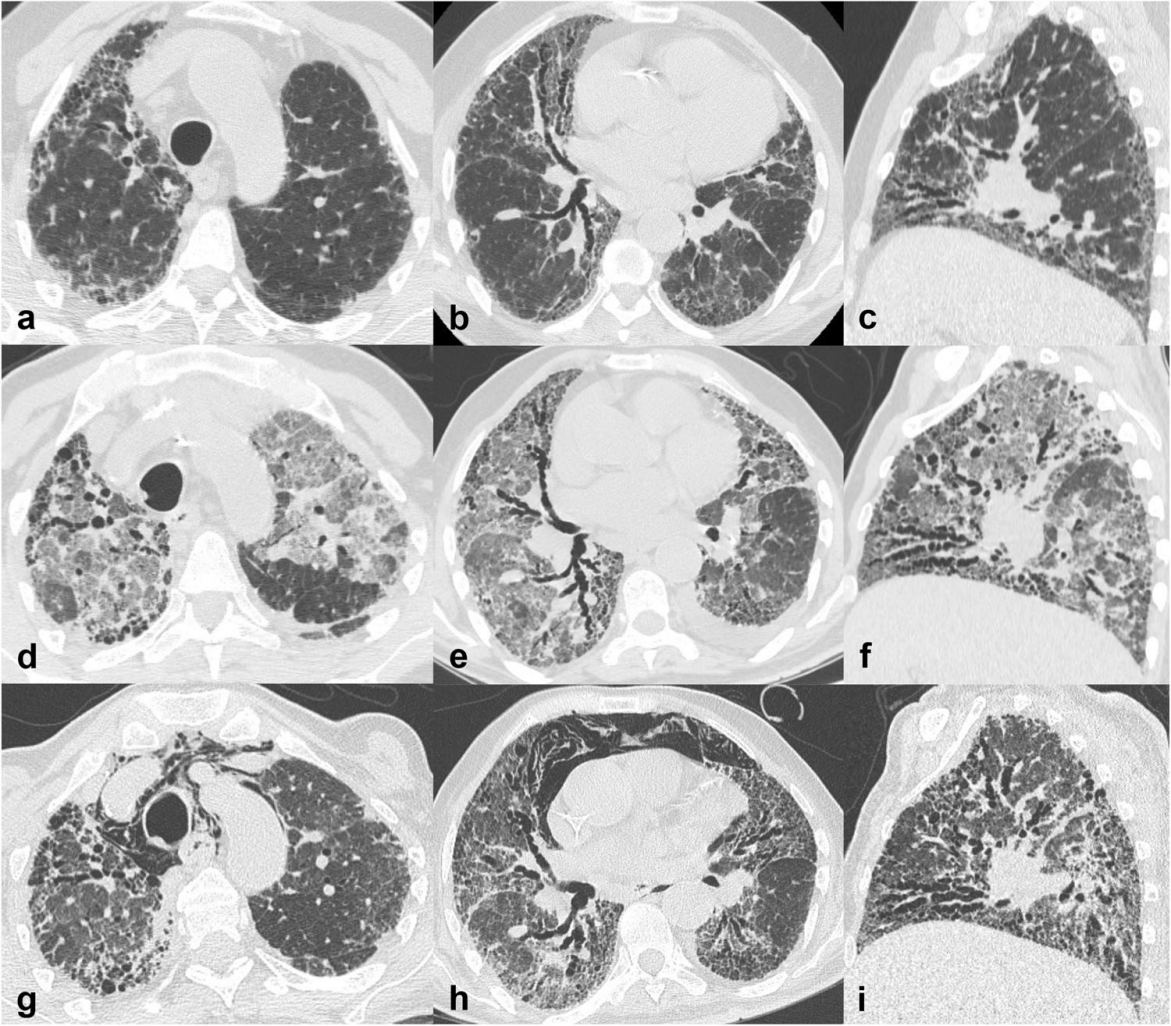
Acute exacerbation of idiopathic pulmonary fibrosis (IPF). Axial and sagittal computed tomography (CT) images (**a**–**c**) depicting a usual interstitial pneumonia (UIP) pattern with the typical peripheral predominant distribution, in the mid-lower posterior zones and antero-superior zones of the lungs (**c**). Honeycombing is evident in the anterior segment of the right upper lobe (**a**). Axial and sagittal CT images (**d**–**f**) performed 15 months later in the emergency department for acute respiratory distress of the patient show the appearance of almost diffuse areas of ground-glass opacity in both lungs, mostly in the areas relatively spared by fibrosis, suggestive of acute exacerbation. After 40 days from the acute phase, a new chest CT scan was requested for persisting chest pain. Axial and sagittal CT images (**g**–**i**), analysed at the same anatomic levels as before, demonstrate the presence of spontaneous pneumomediastinum, explained by the alveoli rupture and interstitial emphysema due to distress; interesting to note the evident progression of pulmonary fibrosis respect to the previous exam, which is a common occurrence after an acute exacerbation

Acute exacerbations (AEs) have a relatively common incidence in IPF patients (5–19% per year), but they can develop in any FLD, causing high mortality. AE is defined as acute respiratory deterioration lasting less than 1 month, with new ground-glass opacities and consolidation appearing on a background of FLD on CT, after cardiac failure or fluid overload have been ruled out [22, 23] (Fig. 7). Radiologists must be aware of the potential occurrence of AEs, especially in the emergency setting, to allow prompt recognition and management; acute pulmonary embolism and pulmonary infections should also be considered in differential diagnosis [22].

Pulmonary hypertension (PH) can complicate FLDs and reduce patients’ overall survival; radiologists should look for CT signs of PH, as the increased main pulmonary artery/ascending aorta diameters ratio (> 0.9), particularly in cases at higher risk (e.g., IPF, systemic sclerosis) [24, 25].

FLDs, particularly IPF and CTD, are associated with an increased incidence of lung cancer, which is most likely to occur near or within fibrotic regions; close monitoring of pulmonary nodules and interdisciplinary evaluation are required for the correct management [26].

### Interdisciplinary cooperation: ILD-board

Ideally, the diagnosis of ILDs is based on interdisciplinary discussions involving radiologists, pneumologists, rheumatologists and pathologists. Various studies have highlighted the positive effects of ILD boards, with an increase in the level of diagnostic agreement and confidence, especially for non-IPF diseases and non-expert centres [27–29]. ILD-boards have a significant impact on final diagnosis, pharmacological or non-pharmacological therapies, with management changes in up to 50% of patients [30, 31]. Lung biopsies are usually recommended by the ILD board when clinical context and radiologic patterns are indeterminate or discordant, emphasising the pivotal role of the radiologist [31].

## Summary statement

CT with high-resolution technique is the recommended imaging modality to correctly recognise signs, patterns, and distribution of pulmonary fibrosis. Systematic interpretation of CT pattern according to the current international guidelines leads to a non-invasive, accurate diagnosis of FLDs or to narrow down the differential diagnosis in the context of interdisciplinary discussion. Biopsy is recommended in indeterminate and discordant cases.

FLDs may show progressive behaviour and reduced survival. Individuals with ILAs might also be at increased risk of disease progression. The major task is to detect these subjects and to direct them to the correct management. An early diagnosis of fibrosis and prompt identification of disease progression are crucial to grant antifibrotic treatment to patients showing a progressive phenotype. FLD patients may also develop complications such as AEs, which are commonly associated with a worsening prognosis.

In this field, radiologist plays a pivotal role. Careful comparison with previous CT examinations is essential to assess progression and complications. The use of reconstructions to provide information on fibrosis distribution, change in pattern and increased extent of lung fibrosis should be recommended. Final diagnosis and therapeutic decisions should be achieved in collaboration with pulmonologists, rheumatologists, and pathologists, working together to improve FLD patients’ management.

## Patient summary

Patients with clinico-functional suspicion of pulmonary fibrosis, as well as those at high risk due to predisposing diseases or family history, should undergo a chest CT with high-resolution technique to identify as early as possible signs of lung fibrosis. Fibrotic lung diseases may show progressive behaviour, leading to reduced survival. Thus, early diagnosis is crucial to grant prompt antifibrotic treatment. Radiologists have a pivotal role not only in the early identification and non-invasive diagnosis of fibrosis but also in the evaluation of disease progression and complications. Interdisciplinary cooperation with pulmonologists, rheumatologists, and pathologists is mandatory for accurate patient management.

## Supplementary information

Electronic Supplementary Material

## Abbreviations

AE(s): Acute exacerbation(s)
ALAT: Asociación Latinoamericana de Tórax
ATS: American Thoracic Society
BMI: Body mass index
CT: Computed tomography
CTD(s): Connective tissue disease(s)
fHP: Fibrotic hypersensitivity pneumonitis
FLD(s): Fibrotic lung disease(s)
fNSIP: Fibrotic nonspecific interstitial pneumonia
FOV: Field of view
ILA(s): Interstitial lung abnormality(es)
ILD(s): Interstitial lung disease(s)
IPF: Idiopathic pulmonary fibrosis
JRS: Japanese Respiratory Society
PFT(s): Pulmonary function test(s)
PH: Pulmonary hypertension
PPF: Progressive pulmonary fibrosis
UIP: Usual interstitial pneumonia
