# Supplementary material for: ESR Essentials: imaging in fibrotic lung diseases—practice recommendations by the European Society of Thoracic Imaging
Source: Eur Radiol. 2024 Sep 7;35(4):2245–55. doi: 10.1007/s00330-024-11054-2 (PMC11914337; doi:10.1007/s00330-024-11054-2)
Supplement: Supplementary file 1 — Electronic Supplementary Material [file 330_2024_11054_MOESM1_ESM.pdf]

ESR Essentials: imaging in fibrotic lung diseases–practice recommendations by the European Society of Thoracic Imaging

Electronic Supplementary Material

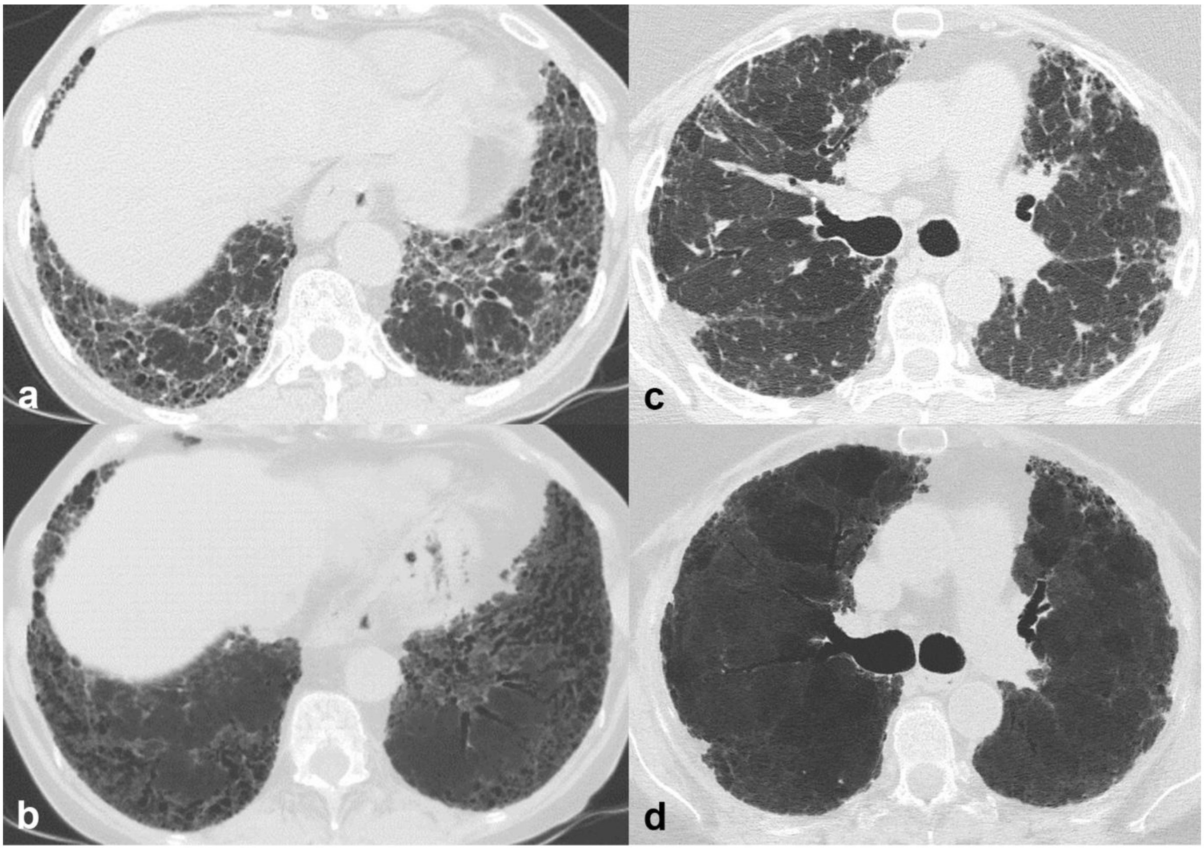

Figure S1: Usefulness of the minimum intensity projection (MinIP) reconstructions. Axial computed tomography (CT) images (**a**, **b**) showing the easy recognition on the MinIP image of peripheral traction bronchiectasis in the background of reticular pattern in the lung bases (**b**). MinIP facilitates the exclusion of honeycombing and the diagnosis of a probable usual interstitial pneumonia (UIP) pattern. Axial CT images (**c**, **d**) showing the added value of MinIP in identifying the 'three-density pattern' in an inspiratory scan (**d**).

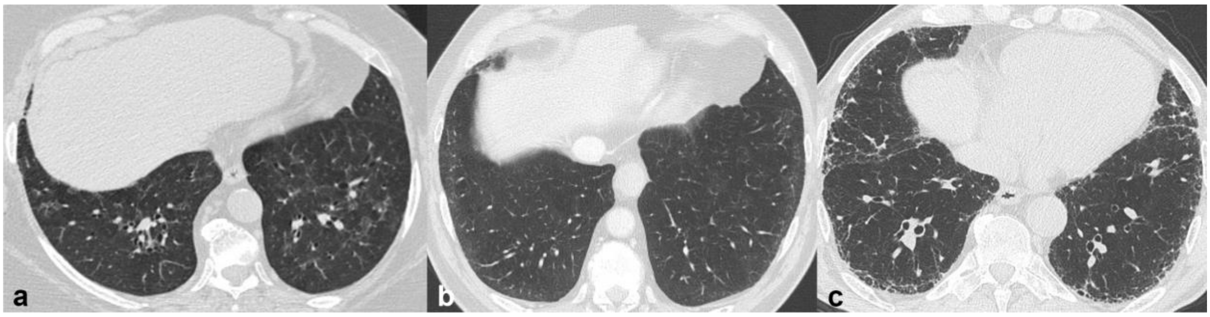

Figure S2: Interstitial lung abnormalities (ILAs) subcategories in three asymptomatic patients. Non subpleural ILA (**a**), with mild peribronchovascular ground-glass in the lower lobes. Subpleural non fibrotic ILA (**b**), with mild subpleural non-dependent ground-glass opacity in both lung bases. Subpleural fibrotic ILA (**c**), characterised by subpleural mild irregular thickening of intralobular and interlobular septa with slight distal bronchiolectasis in the lung bases. The computed tomography (CT) pattern was classified as probable usual interstitial pneumonia (UIP), and a multidisciplinary diagnosis of early idiopathic pulmonary fibrosis (IPF) was done. Antifibrotic treatment was initiated.
